# Adaptive versus maladaptive cardiac remodelling in response to sustained β-adrenergic stimulation in a new ‘ISO on/off model’

**DOI:** 10.1371/journal.pone.0248933

**Published:** 2021-06-17

**Authors:** Stefanie Maria Werhahn, Julia S. Kreusser, Marco Hagenmüller, Jan Beckendorf, Nathalie Diemert, Sophia Hoffmann, Jobst-Hendrik Schultz, Johannes Backs, Matthias Dewenter

**Affiliations:** 1 Institute of Experimental Cardiology, Heidelberg University Hospital, Heidelberg, Germany; 2 DZHK (German Centre for Cardiovascular Research), Partner Site, Heidelberg/Mannheim, Germany; 3 Department of Cardiology and Pneumology, University Medicine Göttingen, Göttingen, Germany; 4 DZHK (German Centre for Cardiovascular Research), Partner Site, Göttingen, Germany; 5 Department of Cardiology, Angiology and Pneumology, Heidelberg University Hospital, Heidelberg, Germany; 6 Department of General Internal Medicine and Psychosomatics, University of Heidelberg, Heidelberg, Germany; Scuola Superiore Sant’Anna, ITALY

## Abstract

On the one hand, sustained β-adrenergic stress is a hallmark of heart failure (HF) and exerts maladaptive cardiac remodelling. On the other hand, acute β-adrenergic stimulation maintains cardiac function under physiological stress. However, it is still incompletely understood to what extent the adaptive component of β-adrenergic signaling contributes to the maintenance of cardiac function during chronic β-adrenergic stress. We developed an experimental catecholamine-based protocol to distinguish adaptive from maladaptive effects. Mice were for 28 days infused with 30 mg/kg body weight/day isoproterenol (ISO) by subcutaneously implanted osmotic minipumps (‘ISO on’). In a second and third group, ISO infusion was stopped after 26 days and the mice were observed for additional two or seven days without further ISO infusion (‘ISO off short’, ‘ISO off long’). In this setup, ‘ISO on’ led to cardiac hypertrophy and slightly improved cardiac contractility. In stark contrast, ‘ISO off’ mice displayed progressive worsening of left ventricular ejection fraction that dropped down below 40%. While fetal and pathological gene expression (increase in *Nppa*, decrease in *Myh6*/*Myh7* ratios, increase in *Xirp2*) was not induced in ‘ISO on’, it was activated in ‘ISO off’ mice. After ISO withdrawal, phosphorylation of phospholamban (PLN) at the protein kinase A (PKA) phosphorylation site Ser-16 dropped down to 20% as compared to only 50% at the Ca^2+^/Calmodulin-dependent kinase II (CaMKII) phosphorylation site Thr-17 in ‘ISO off’ mice. PKA-dependent cardioprotective production of the N-terminal proteolytic product of histone deacetylase 4 (HDAC4-NT) was reduced in ‘ISO off’ as compared to ‘ISO on’. Taken together, these data indicate that chronic ISO infusion induces besides maladaptive remodelling also adaptive PKA signalling to maintain cardiac function. The use of the ‘ISO on/off’ model will further enable the separation of the underlying adaptive from maladaptive components of β-adrenergic signalling and may help to better define and test therapeutic targets downstream of β-adrenergic receptors.

## Introduction

Heart failure (HF) is a main cause of morbidity and mortality worldwide [1]. HF is a clinical syndrome that is caused by a variety of inherited or acquired factors and is characterized by structural cardiac abnormalities and impaired cardiac function [2]. One characteristic hallmark of HF is the pronounced activation of the sympathetic nervous system leading to sustained stimulation of β-adrenergic receptors and multiple downstream effects [3,4]. In experimental cardiology, forced sympathetic stimulation through application of synthetic catecholamines is widely used to induce cardiac remodelling and eventually HF. The administration of isoproterenol (ISO) is one of the most frequently used synthetic catecholamines to elicit cardiac remodelling and HF in animal models [5–7]. ISO is a non-selective agonist of β-adrenergic receptors that acutely exerts positive inotropic and chronotropic effect [8]. Chronic ISO exposure is reported to lead to adverse cardiac remodelling with subsequent deterioration of cardiac function. It is suggested that in this setting decompensated HF is preceded by adaptation and compensated cardiac hypertrophy along with improved diastolic and systolic function [5,6]. Despite the frequent use of ISO, experimental protocols to induce myocardial changes by ISO administration differ widely. ISO effects seem to depend on the mode of administration, the administered dose and the duration of treatment but also on genetic factors [5,9]. Considering the multiple downstream signalling pathways of β-adrenergic receptors, the heterogenous use might result differentially in activation of adaptive or maladaptive signalling cascades. In this regard, activation of protein kinase A (PKA) in an acute setting seems rather to be adaptive and activation of Ca^2+^/Calmodulin-dependent kinase II (CaMKII) in a chronic setting rather to be maladaptive [10–12]. However, the relative contribution of the adaptive component of β-adrenergic signalling in the chronic and sustained setting of β-adrenergic stimulation is unclear. The primary purpose of this study was to identify the existence of a potentially adaptive component of β-adrenergic signalling in a chronic ISO model and to start to delineate the nature of this component. For this purpose, we established a new protocol that allows to further elucidate this Janus-head nature of ISO on cardiac function. We show that continuous ISO-infusion results in compensated cardiac remodelling characterized by hypertrophy and maintained cardiac function that turns to progressive, decompensated HF when ISO administration is stopped. As possible underlying mechanism, we found a more pronounced drop in the activity of PKA relative to CaMKII.

## Materials and methods

### Animals

The experiments were carried out using 8 to 12 weeks-old male C57BL/6N mice purchased from Charles River. All animals were fed a standard diet and were maintained on a 12-h light and dark cycle at a room temperature of 22 ± 2°C. All experimental procedures were reviewed and approved by the Institutional Animal Care and Use Committee at the Regierungspräsidium Karlsruhe, Germany.

### ISO-induced cardiac remodelling

To induce cardiac remodelling, mice were subjected to chronic β-adrenergic stress using continuous ISO (Isoproterenol Bitartrate, I2760; Sigma Aldrich) infusion. ISO which was administered at a dosage of 30 mg/kg body weight/day for 26 (‘ISO off’) or 28 days (‘ISO on’), respectively, via subcutaneously implanted osmotic minipumps (Alzet Osmotic Pump Model 2004, Durect Corporation). In order to prevent chemical decay, ISO was dissolved in a carrier solution (150 mmol/l NaCl with the addition of 0,1 mmol/l ascorbic acid) that also served as control. For the implantation of the pump, animals were anesthetized with Ketamine/Xylazine (120 mg/kg BW Ketamine and 16 mg/kg BW Xylazine). After incising of the scin (1 cm) between the shoulder blades on the back of the animal, the osmotic pump was inserted into the subcutaneous tissue and the wound was closed with two stitches (6–0 Prolene). After 26 days of ISO treatment, the pump was removed in two groups of mice (‘ISO off short’ and ‘ISO off long’). The half-life of subcutaneously administered ISO is about 2 hours [13]. In the ‘ISO off short’ group organ harvesting was performed together with the ‘ISO on’ group on day 28 post pump implantation and in the ‘ISO off long’ group organ harvesting was performed on day 33.

### Echocardiography

Transthoracic micro-echocardiography was conducted using a VisualSonics Vevo 2100 echocardiography setup equipped with a MS400 transducer. The investigator was blinded to the treatment and control groups. Mice were shaved and anaesthetized for each ultrasound with 2 vol% isoflurane, and light isoflurane anaesthesia (1–1.5 vol%) was maintained during the measurement. Left ventricular (LV) parasternal long axis and short axis views at the mid-papillary muscle level were obtained. Analysis was performed with VisualSonics Vevo Lab software, applying LV tracing measurements. A minimum of five consecutive beats were used to measure ejection fraction (EF), fractional shortening (FS) and left ventricular mass (LVM) from short axis M-mode images applying the following calculations: EF = 100 * ((LV Vol;d–LV Vol;s) / LV Vol;d) with LV Vol;d = ((7.0 / (2.4 + LVID;d)) * LVID;d^3^ and LV Vol;s = ((7.0 / (2.4 + LVID;s)) * LVID;s^3^; FS = 100 * ((LVID;d–LVID;s) / LVID;d); LVM = 1.053 * ((LVID;d + LVPW;d + IVS;d)3 –LVID;d3) * 0.8. Echocardiography was performed in all mice on days 0 (pre pump implantation), 14, 26 and 28 as well as on day 33 in ‘ISO off long’ mice and respective controls.

### Pressure-Volume-loop (PV-loop) analyses

Closed chest PV-measurements were performed by left ventricular catheterization as previously described [14]. After initiation of general anaesthesia with propofol 1% (0.002 ml/g bodyweight, retroorbital injection) and isoflurane anaesthetic gas, mice were intubated and ventilated with a rodent ventilator (MiniVent Type 845, Harvard Apparatus). Anaesthesia was maintained with isoflurane 2 vol%. Briefly, the right carotid artery was exposed and a 1.4 French pressure-conductance catheter with 4.5 mm electrode spacing (FTM-1212B-4518, Scisense) was retrogradely advanced into the left ventricle. After optimization of catheter tip position, anaesthesia was reduced to 0.5 vol% isoflurane to provide physiological hemodynamic readings. Steady-state PV measurements were obtained and registered using the MPVS-300 system (Millar Instruments) and Chart 5.0 with PVAN 3.5 analysis software (ADInstruments). Pressure-volume loops were only performed in Sham (control), ‘ISO on’ and ‘ISO off short’ mice.

### Organ harvesting

Hearts and lungs were removed and weighed promptly. Relative heart and lung weights were calculated as ratios to body weight or tibia length. Left ventricles were transversely dissected, and parts of the left ventricles were quickly frozen in liquid nitrogen for protein and RNA isolation and analysis. Tissue samples for histological staining were fixed in 4% paraformaldehyde and embedded in paraffin.

### Cardiac histology

Paraffin-embedded tissue samples were longitudinally cut into 5 μm thick cardiac sections, deparaffinized and stained with haematoxylin and eosin (H&E) or Sirius Red. H&E staining was performed using the Carl Roth H&E fast staining kit (9194.1) according to the manufacturer’s instructions. For fibrosis analysis, sections were stained with Picro-Sirius Red Solution (Biozol) for 30 min, washed in two changes of acidified water (1% acetic acid) and dehydrated in an ascending series of alcohol. Sections were mounted with EUKITT neo (Kindler). Microscopic images were captured with the ZEISS Axio Scan.Z1 slide scanner. Cardiomyocyte size was assessed on H&E-stained sections. Cross-sectional areas of 150–200 randomly chosen transversely cut cardiomyocytes from each slide were measured using ImageJ. Whole transversal Sirius Red stained sections were used to quantify the fibrotic area. The Sirius Red stained area was quantified using ImageJ by applying grayscale threshold in RGB stacks and normalized to total area. All data were analysed by two observers blinded to group allocation.

### RNA isolation and quantitative real-time PCR (qPCR)

Total RNA was isolated from ventricular tissue using TRIzol (Invitrogen, Germany). Total RNA was digested with DNase, and cDNA synthesis from 500 ng of RNA was carried out using the Invitrogen SuperScript First-Strand Synthesis System for RT-PCR. qPCR was performed on a Lightcycler 480 system II (Roche) using SYBR Green (Roche) and data was analyzed using LinRegPCR software. The following primers were used: *Gapdh* (housekeeping gene): 5’-ggtggacctcatggcctaca-3’ and 5’-ctctcttgctcagtgtccttgct-3’; *Nppa*: 5’-cacagatctgatggatttcaaga-3’ and 5’-cctcatcttctaccggcatc-3’; *Myh6*: 5-’ggagtgcttcgtgcctgatg-3’and 5’-gaacttgggtgggttctgct-3’; *Myh7*: 5’-cgcatcaaggagctcacc-3’and 5’-ctgcagccgcagtaggtt-3’; *Xirp2*: 5’-ggtaccaggcagctgtttca-3’ and 5’-aaaggcgttgcaggttgaag-3’.

### Western blot analysis

Cardiac extracts preparation and Western blot analysis were carried out as described in an earlier publication [14]. Antibodies for immunoblotting were used as follows: mouse anti-beta-tubulin (Sigma), mouse anti-phospholamban (PLN) (Upstate), rabbit anti-phospho-PLN (Thr-17) (Upstate), rabbit anti-phospho-PLN (Ser-16) (Upstate), goat anti-HDAC4 (SantaCruz, N18).

### Statistical analysis

Results are reported as mean±SD. Statistical analysis was performed with GraphPad Prism Software Package version 9.0.1. Normality tests (Anderson-Darling, D’Agostino-Pearson, and Shapiro-Wilk tests) were performed on each dataset. Groups of datasets with a Gaussian distribution were compared by one-way ANOVA followed by Turkey multiple comparison test (for comparison of more than two groups) or unpaired t-test (for comparison of two groups). Groups of datasets with non-normal distribution were compared by Kruskal-Wallis followed by Dunn’s multiple comparison test. A value of *P*<0.05 was considered statistically significant.

## Results

### The ‘ISO on/off model’

**Fig 1:**
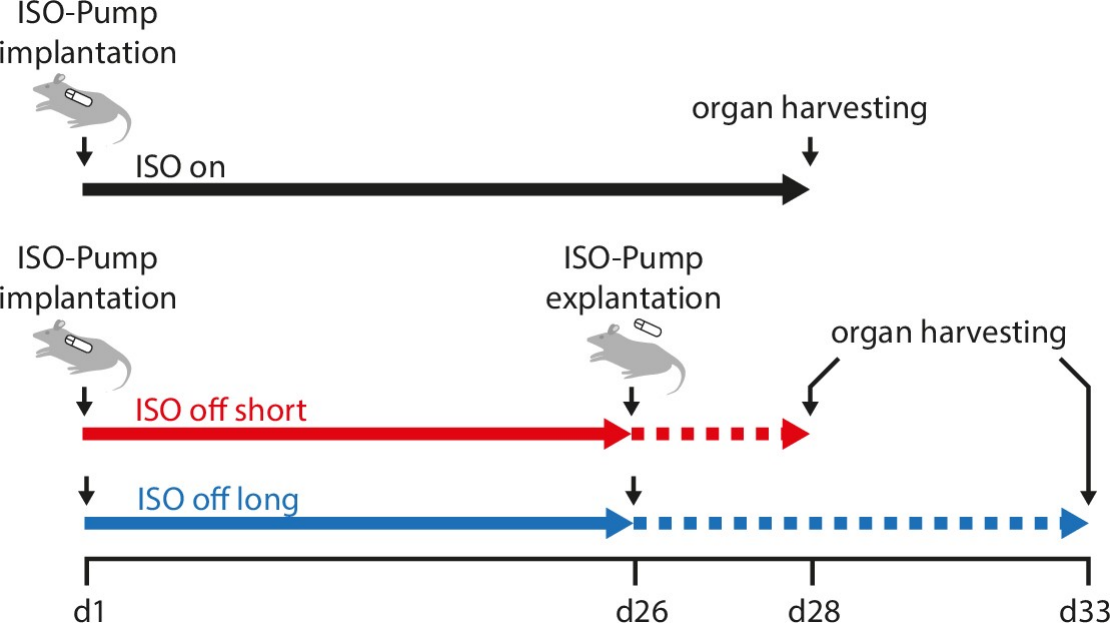
Experimental protocol. ISO was administered in male C57BL/6N mice via subcutaneously implanted osmotic minipumps at a dose of 30 mg/kg body weight/day. The ‘ISO on’ mice received ISO until the end of the experiment on day 28 when organ harvesting took place. In ‘ISO off’ mice, the pump was explanted on day 26 to analyse the effects of catecholamine withdrawal for two days (‘ISO off short’) and seven days (‘ISO off long’) after pump removal.

‘ISO on’ mice were infused with ISO s.c. for 28 days via osmotic minipumps. In ‘ISO off’ mice the osmotic minipump was explanted on day 26. The effect of ISO withdrawal was studied at days 2 and 7 after pump removal (‘ISO off short’ and ‘ISO off long’). Cardiac function was assessed by echocardiography on day 0 (pre), 14, 26, 28 and day 33 in ‘ISO off long’, respectively. Organ harvesting was done on day 28 (‘ISO on’, ‘ISO off short’) and day 33 (‘ISO off long’). This new protocol is named ‘ISO on/off model’ and depicted in **Fig 1.**

### ISO administration leads to reversible cardiac hypertrophy

**Fig 2:**
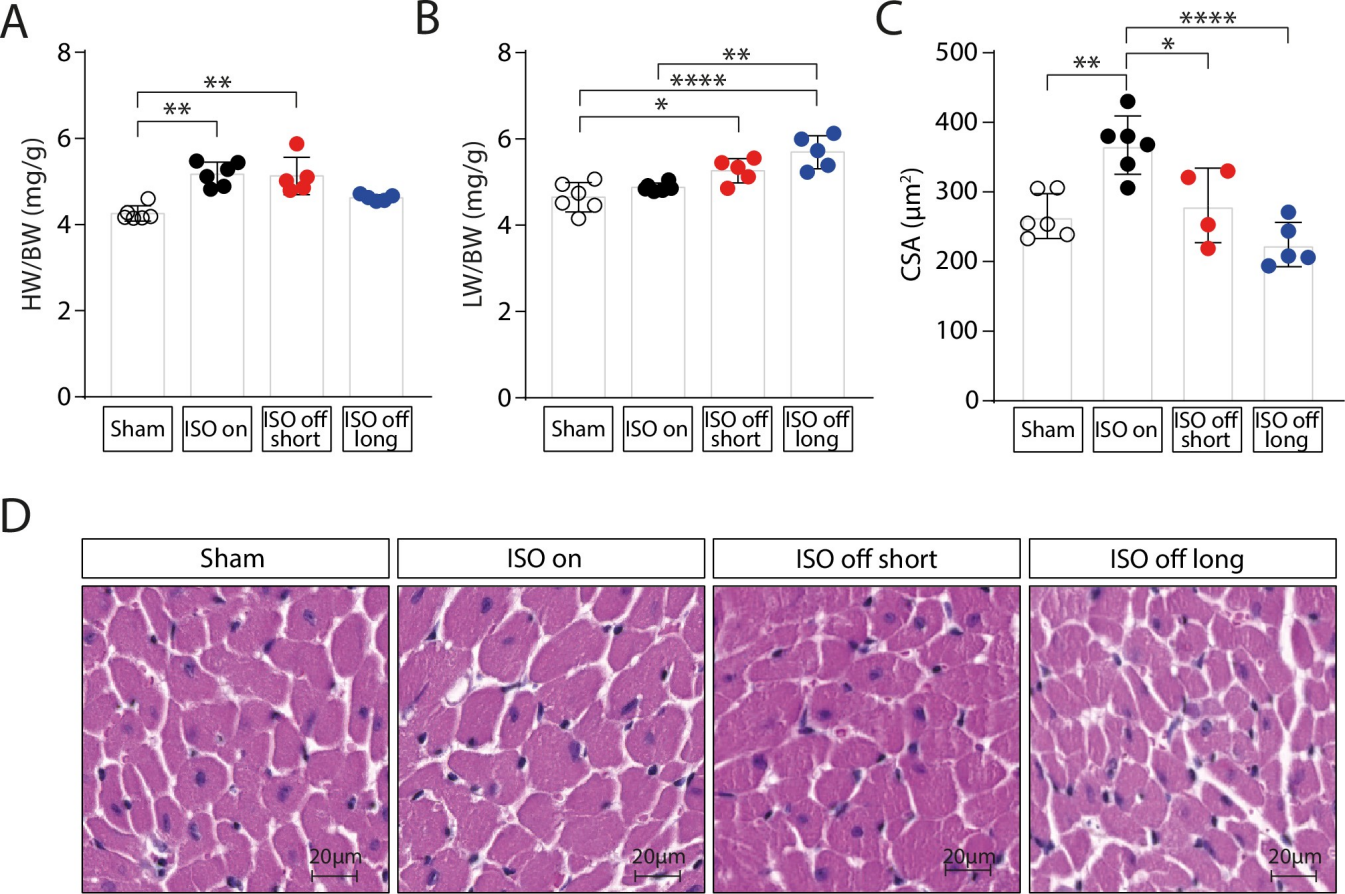
ISO leads to cardiac hypertrophy. (A) Heart Weight/Body Weight (HW/BW) ratios, (B) Lung Weight/Body Weight (LW/BW) ratios and (C) Cardiomyocyte Cross-Sectional Area (CSA) quantification from haematoxylin and eosin stained cardiac sections in Sham (control), ‘ISO on’, ‘ISO off short’ and ‘ISO off long’ mice at the endpoint of the study. (D) Representative haematoxylin and eosin images of cardiac sections. Sham (control): n = 6; ‘ISO on’: n = 6; ‘ISO off short’: n = 5; ‘ISO off long’: n = 5. 150–200 cardiomyocyte CSAs were measured for each individual. Data are presented as mean ± SD. *P < 0.05; **P < 0.01; ***P < 0.001; ****P < 0.0001.

Four weeks of sustained ISO infusion (‘ISO on’) caused notable cardiac hypertrophy, as indicated by increased ratios of heart weight/body weight (HW/BW) (**Fig 2A**) and heart weight/tibia length (HW/TL) (**S1 Fig**). Upon ISO withdrawal we observed a progressive regression of cardiac hypertrophy within 7 days (**Figs 2A and S1**). Lung weights were increased in the ‘ISO off short’ and more pronounced in the ‘ISO off long’ group, indicating increasing pulmonary congestion (**Fig 2B**). Histological analysis of cardiac sections confirmed the induction of cardiomyocyte hypertrophy under chronic ISO administration, which was reversible after termination of ISO administration (**Fig 2C and 2D**). Analysis of cardiac Sirius Red stainings revealed only a mild and non-significant effect of ISO infusion on cardiac fibrosis (**S2 Fig**).

### Cardiac dysfunction upon ISO withdrawal

**Fig 3:**
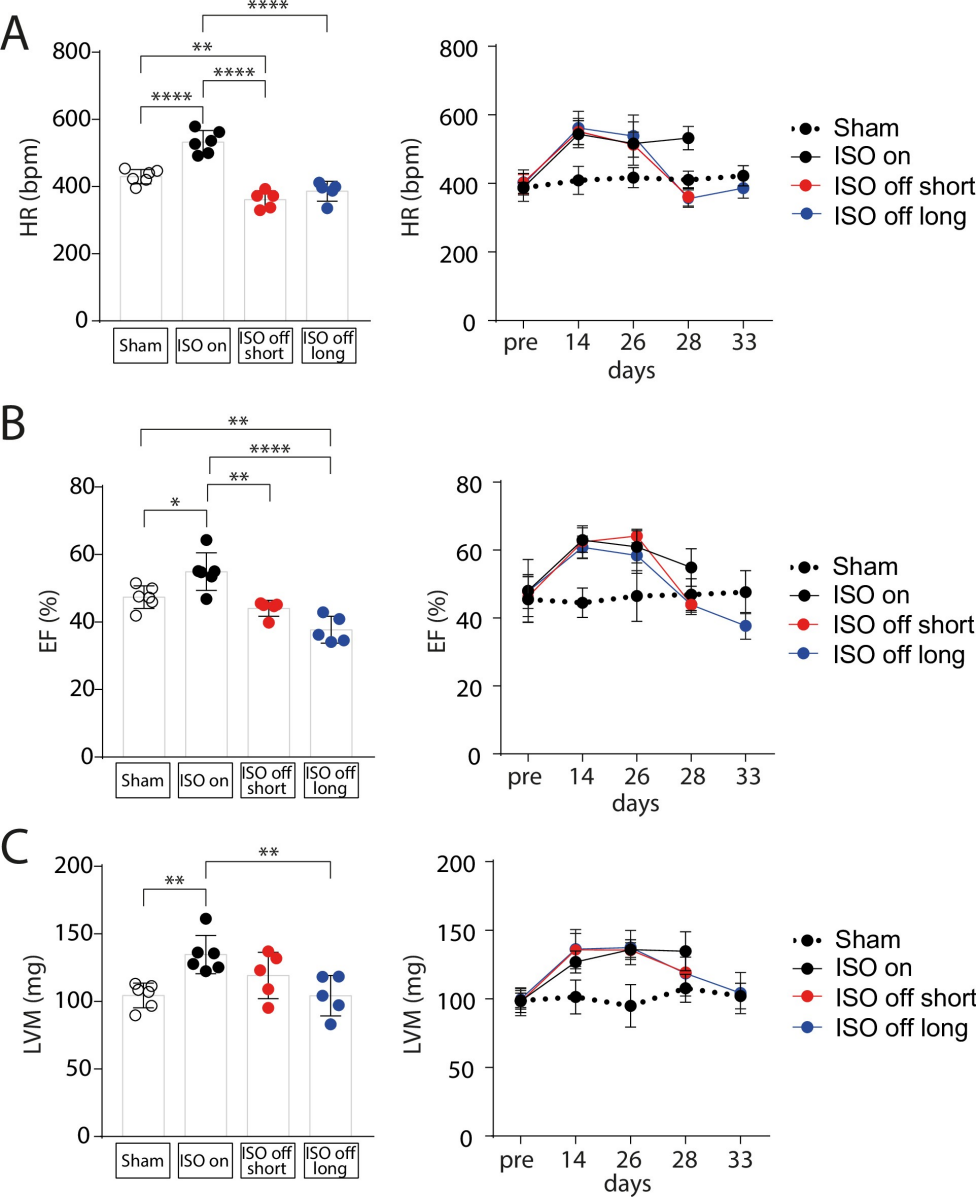
Cardiac function is maintained under ISO and deteriorates upon ISO withdrawal. Echocardiographic measurement of (A) Heart Rate (HR) in beats per minute (bpm), (B) left ventricular Ejection Fraction (EF) in % and (C) Left Ventricular Mass (LVM) in mg at the endpoint and in the time course of the study in Sham (control), ‘ISO on’, ‘ISO off short’ and ‘ISO off long’ mice. Transthoracic echocardiography was performed on day 0 (pre pump implantation) 14, 26, 28 in all mice and on day 33 in ‘ISO off long’ and respective control mice. Sham (control): n = 6; ‘ISO on’: n = 6; ‘ISO off short’: n = 5; ‘ISO off long’: n = 5. Data are presented as mean ± SD. *P < 0.05; **P < 0.01; ***P < 0.001; ****P < 0.0001.

In ‘ISO on’ mice, transthoracic echocardiography revealed a significant increase in heart rate (HR) (**Fig 3A**), left ventricular ejection fraction (EF) (**Fig 3B**) and fractional shortening (FS) (**S3 Fig**). Upon termination of ISO, we observed a rapid worsening of left ventricular contractility that dropped below the level of the control group in ‘ISO off long’ mice (**Figs 3B and S3**). Heart rate, however, normalized to the level of control mice on day 7 of ISO withdrawal after an initial drop after pump explantation, indicating that the decline in contractile function cannot merely be explained by beta-adrenoceptor desensitization (**Fig 3A**). Pressure volume measurements confirmed the functional improvement induced by ISO and the shift towards cardiac dysfunction in ‘ISO off’ mice (**S4 Fig**). In accordance with the morphological and histological findings (**Fig 2**), echocardiographic analysis of left ventricular mass (LVM) also showed reversible cardiac hypertrophy induced by ISO administration (**Fig 3C**).

### Fetal and pathological gene expression upon ISO withdrawal

**Fig 4:**
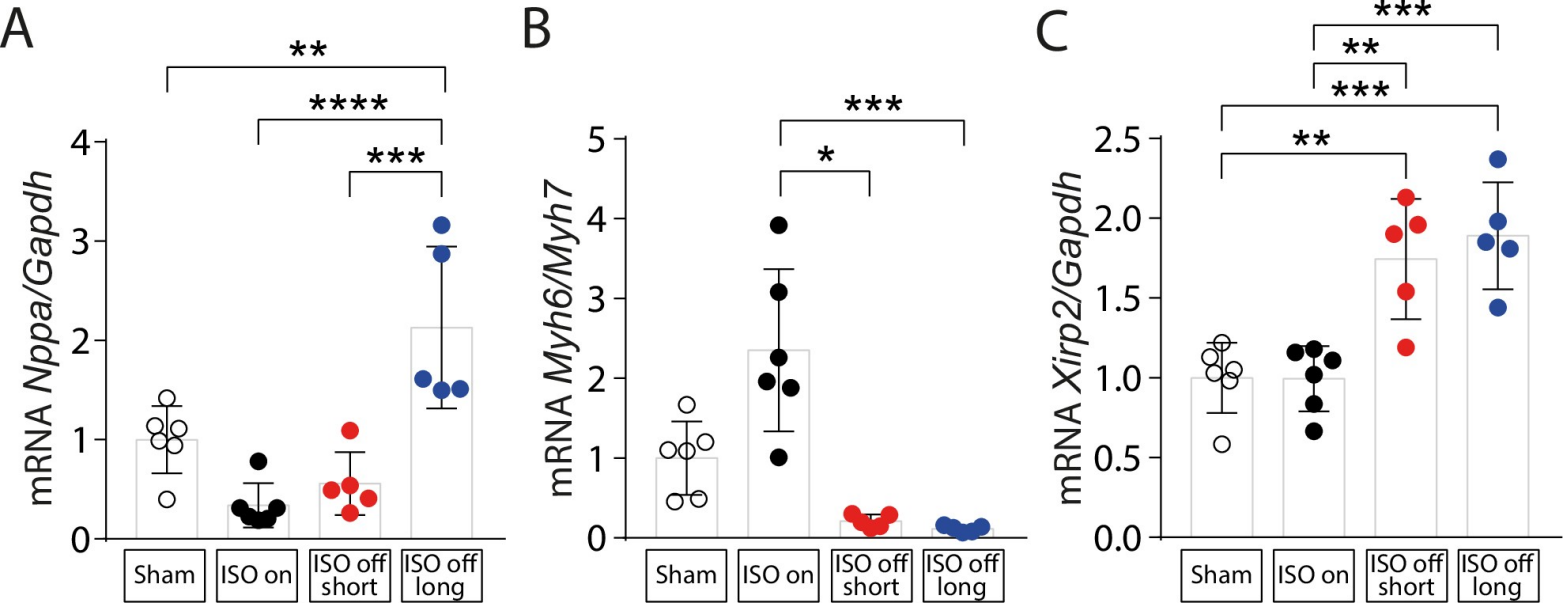
Activation of fetal and pathological genes upon termination of continuous ISO stimulation. Quantitative RT–PCR measurements of (A) *Nppa* expression normalized to *Gapdh* (B) *Myh6*/*Myh7* ratios and (C) *Xirp2* expression normalized to *Gapdh* in cardiac tissue from Sham (control), ‘ISO on’, ‘ISO off short’ and ‘ISO off long’ mice at the endpoint of the study. Sham (control): n = 6; ‘ISO on’: n = 6; ‘ISO off short’: n = 5; ‘ISO off long’: n = 5. Data are presented as mean ± SD. *P < 0.05; **P < 0.01; ***P < 0.001; ****P < 0.0001.

Whereas ‘ISO on’ mice did not show an activation of the fetal gene program as detected by *Nppa* mRNA levels, *Nppa* expression was activated in ‘ISO off’ mice (**Fig 4A**). To judge the switch to pathological gene expression, we measured the ratio of *Myh6/Myh7* and found that it was increased in ‘ISO on’ but decreased in ‘ISO off’ mice (**Fig 4B**), suggesting that ISO treatment caused cardioprotection. In addition, expression of the MEF2 target gene Myomaxin (*Xirp2*) was induced in the ‘ISO off’ groups, further indicating the switch to pathological gene expression upon ISO withdrawal (**Fig 4C**).

### ISO withdrawal results in a stronger drop in PKA than CaMKII activity

**Fig 5:**
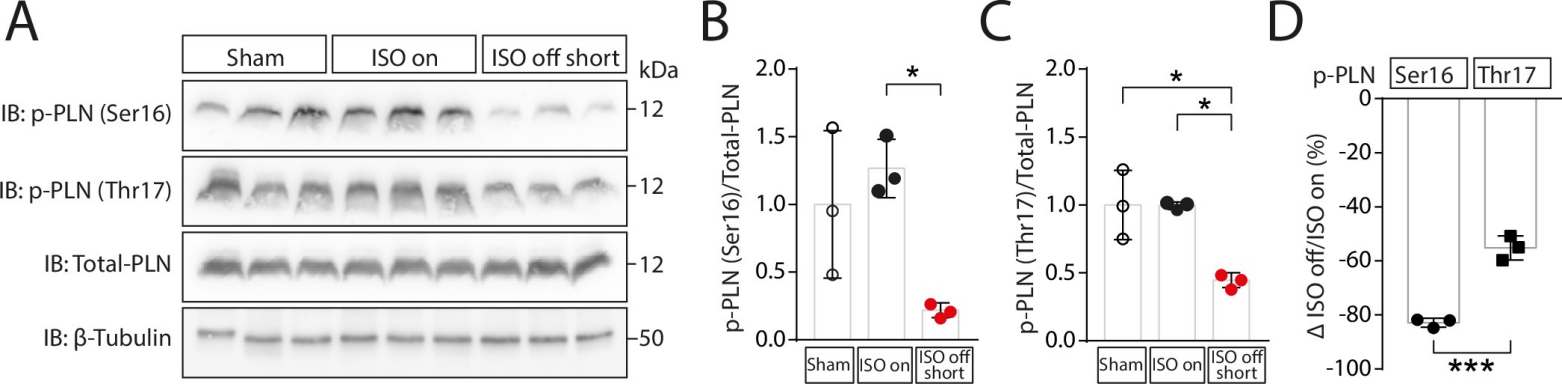
ISO-induced phosphorylation of PLN. (A) Western Blots images and (B) quantification of phospholamban (PLN) phosphorylation at the PKA site Ser16 (pPLN-Ser16) and (C) at the CaMKII site Thr17 (pPLN-Thr17) in cardiac tissue from Sham (control), ‘ISO on’ and ‘ISO off short’ mice on day 28. (D) Ratio of pPLN-Ser16/P-PLN-Thr17 in ‘ISO on’ versus ‘ISO off short’. Data are presented as mean ± SD. *P < 0.05. The uncropped images of the immunoblots are presented in S5 Fig.

We then analysed in cardiac extracts the phosphorylation status of phospholamban (PLN) that contains different phosphorylation sites for two downstream kinases of β-adrenergic receptors, protein kinase A (PKA) and Ca^2+^/Calmodulin-dependent Kinase II (CaMKII), that play opposite roles with respect to adaptation and maladaptation [3,10,15,16]. Western blot analysis of cardiac tissue from ‘ISO on’ mice revealed a drastic drop of phosphorylation of PLN at the PKA site Ser-16 (pPLN-Ser16) but only a modest loss of phosphorylation at the CaMKII site Thr-17 (pPLN-Thr17) (**Fig 5A–5D**). Somewhat surprising, these data suggest that in the chronic situation of 4 weeks ISO infusion, PKA-mediated phosphorylation relies more on sustained catecholaminergic stimulation than CaMKII-mediated phosphorylation, thereby unmasking that the drop of cardiac function is associated with a relative shift from PKA to CaMKII activity.

### Less production of HDAC4-NT after ISO removal

**Fig 6:**
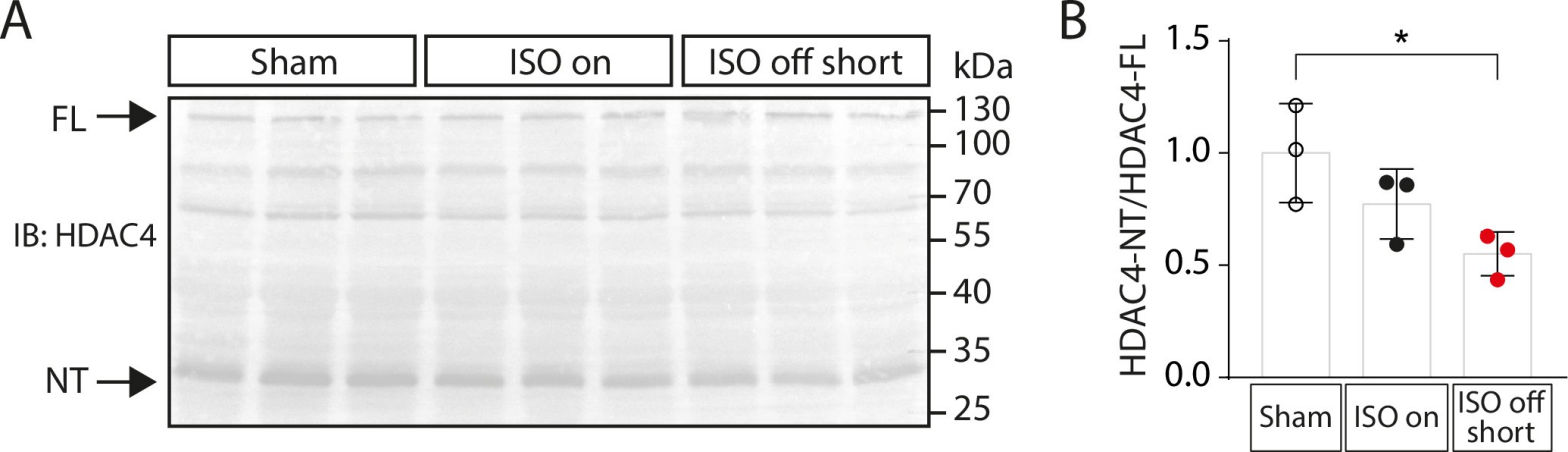
HDAC4-NT in mice after ISO-treatment. (A) Western Blot of HDAC4 full length (FL) and HDAC4-NT and (B) quantification of the HDAC4-NT/FL ratio in cardiac tissue from Sham (control), ‘ISO on’ and ‘ISO off short’ mice on day 28. Data are presented as mean ± SD. *P < 0.05. The uncropped image of the immunoblot is presented in S6 Fig.

The production of an N-terminal proteolytic product of HDAC4 (HDAC4-NT) has been shown to depend on PKA and lipid droplet-associated signalling and to be cardioprotective because it maintains cardiac function and prevents cardiac fatigue [10,15,17,18]. We hypothesized that the drop of PKA activity in ‘ISO off’ mice will lead to a reduction of HDAC4-NT. Indeed, we found a decrease of HDAC4-NT in ‘ISO off’ mice (**Fig 6**). These findings match the increased expression of the MEF2 downstream target Myomaxin in ‘ISO off’ mice (**Fig 4C**), indicating that the decrease of HDAC4-NT causes an activation of pathological MEF2-dependent gene expression in the ‘ISO off’ groups. A loss of PKA-driven HDAC4 proteolysis is therefore associated with ‘ISO on/off’-induced maladaptive cardiac remodelling with subsequent deterioration of cardiac function. It will be interesting to investigate in the future whether the relative loss of HDAC4-NT is causative for cardiac dysfunction in this setting as it is in chronic exercise [10].

## Discussion

**Fig 7:**
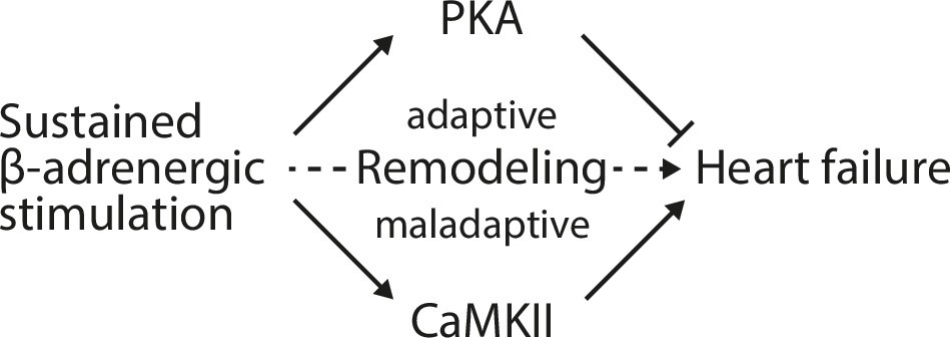
Working model. Sustained β-adrenergic stimulation leads through a balanced regulation of PKA and CaMKII signalling to adaptive and maladaptive remodelling processes and eventually to heart failure. Adaptive PKA signalling can counteract CaMKII-driven maladaptive signalling and its loss leads to overt cardiac dysfunction.

The role of β-adrenergic signalling in the heart has been an area of interest for half a century, since betablockers have been developed as a class of drugs that improve the outcome of HF patients [2]. Nevertheless, it is still puzzling that β-adrenergic signalling can induce both adaptive and maladaptive effects on cardiac function, morphology and metabolism [3,18,19]. Here, we present a new animal model that will help to better dissect this Janus-faced nature of β-adrenergic signalling. Our main findings are that (i) chronic infusion of the non-selective β-adrenergic agonist ISO results in compensated cardiac hypertrophy with slightly improved cardiac function, (ii) ISO withdrawal stops transient catecholamine-induced adaptation and unmasks the chronic maladaptive features resulting in cardiac dysfunction, and (iii) the drop in cardiac function is associated with a strong reduction of PKA activity and the loss of cardioprotective HDAC4-NT production (**Fig 7**).

### Induction of myocardial injury by ISO

Up to now, a plethora of experimental studies using different experimental protocols for ISO-induced cardiac hypertrophy and HF in rodents has been carried out. In all of them, ISO led to cardiac remodelling, which was characterized by various degrees of cardiac hypertrophy, fibrosis, and changes in cardiac function ranging from hypercontractility to severe systolic and diastolic dysfunction [5,6,13,20–31]. The heterogeneity of cardiac phenotypes caused by ISO can methodologically be attributed to the application, the dosage and duration of ISO-administration or to differences in genetic mouse strains, sex or age [7,32,33]. Intermittent β-adrenergic stimulation through subcutaneous or intraperitoneal ISO-injections [5,13,34,35] resulted in more pronounced cardiac fibrosis and dysfunction than continuous ISO-application via subcutaneously implanted osmotic mini-pumps [5,13,16,23,25,30,31,36]. The higher and the longer the ISO-dose administered, the greater was the degree of adverse cardiac remodelling. As evidenced by the present work, the timepoint of functional measurements, organ harvesting and analyses (during ISO-administration or after an ISO-free period) is of particular importance to differentiate short-term effects in the situation of chronic stress [5,24–26].

### Withdrawal of ISO unmasks pathological cardiac remodelling

As expected and consistent with previous publications [13,16,21,27,29,30], ISO elicited moderate cardiac hypertrophy in ‘ISO on’ mice. In most experimental studies using similar experimental protocols [5,13,16,27,29,30], ISO-induced myocardial hypertrophy was associated with cardiac fibrosis, enhanced expression of maladaptive genes like the so-called fetal gene program and even cardiac dysfunction. By contrast and in line with other publications [5,21,25,29], in our study ‘ISO on’ mice presented with maintained cardiac function and without gene expression changes that are typically associated with HF. It therefore seems plausible that part of the hypertrophic response elicited by ISO was mediated by signalling pathways characteristic for adaptation [37]. The deterioration of cardiac function and activation of fetal and pathological genes in ‘ISO off’ mice indicate that ISO indeed induced components of maladaptive remodelling that were masked by counteracting adaptive components that persisted as long ISO was infused. In contrast to some other publications, we observed only a mild effect of ISO on cardiac fibrosis. This finding, however, is in accordance with previous reports demonstrating that ISO has no or only mild pro-fibrotic effects in B57Bl/6 mice [33,38]. We conclude that under ongoing ISO infusion transient adaptive components compensated for maladaptive remodeling. Consequently, ISO withdrawal unmasked pathological cardiac remodelling and led to overt HF.

### Janus-headed nature of β-adrenergic downstream signalling

To start to delineate underlying signalling cascades, we analysed the phosphorylation status of PLN in cardiac extracts as a surrogate for PKA and CaMKII activity [39]. We found that PKA and CaMKII signalling cascades were both maintained under ISO stimulation, but that PKA activity was relatively more reduced than CaMKII activity after ISO withdrawal. Given that PKA in relation to CaMKII is rather involved in adaptation to stress than in maladaptation [11,15,18,40,41], it is reasonable to speculate that the strong loss of PKA activity after withdrawal of ISO is responsible for the induction of cardiac dysfunction [3]. We could recently show that one of the PKA-dependent maintenance factors for cardiac function is HDAC4-NT [10,15,18]. PKA phosphorylates the lipid droplet-associated protein perilipin to induce lipolysis and proteolysis of HDAC4 through the serine protease ABHD5 [18]. The resulting N-terminal part of HDAC4, HDAC4-NT, inhibits gene expression of glycolytic genes and in particular genes that activate the hexosamine biosynthetic pathway with subsequent protein O-GlcNAcylation including STIM1L, which critically contributes to the maintenance of cardiac function [10,17]. Interestingly, HDAC4-NT was reduced in the setting of ISO withdrawal, potentially explaining the transition to HF. Additionally, it has recently been shown that PKA activation triggers nuclear accumulation of HDAC5 by direct phosphorylation or oxidation of HDAC5 or by inhibition of protein kinase D (PKD) [42–46], which can also be attributed to the adaptive PKA effects. Moreover, it has been reported that PKA phosphorylates and inhibits the transcription factor MEF2 directly and thereby can inhibit gene expression programs that have been shown to drive pathological cardiac remodelling [19,47]. Indeed, we observed an increase in the expression of prominent MEF2 target gene Myomaxin (*Xirp2*) only in the ‘ISO off’ groups, indicating a shift towards enhanced MEF2 activation upon ISO withdrawal. It will be interesting to use the ‘ISO on/off” model to determine the relative contribution of the aforementioned mechanisms to the cardioprotective effects of PKA but also to better identify and test the maladaptive components of β-adrenergic signalling such as CaMKII. Surprisingly, in ‘ISO on’ mice sustained β-adrenergic signalling did not lead to detectable activation of CaMKII and it was even further decreased upon ISO removal, questioning whether CaMKII is the main force of maladaptive remodelling or whether it is sufficient to shift the relative balance from PKA to CaMKII activity. To answer whether CaMKII is required for the pathological events in ‘ISO on’ mice that lead to HF in ‘ISO off’ mice, the ‘ISO on/off’ model shall be combined with models of CaMKII inhibition. However, the ‘ISO on/off’ model can also be used to test other candidate pathways in combination with appropriate genetic mouse models.

### Clinical implications

In clinical emergency reality, HF patients with acute decompensation are frequently treated with catecholamines as positive inotropic support [48]. It is well known that sustained intravenous catecholamine treatment besides the acute life-saving effects due to an improvement of the hemodynamic situation is associated with a worse long-term outcome [48]. Thus, it is a clinical challenge to decide when and how long to treat these critically ill patients with positive inotropic support. The ‘ISO on/off’ model may help to identify crucial and druggable components downstream of β-adrenergic receptors. For instance, the blockade of a critical downstream signalling event may help to reduce the maladaptive features of catecholamine treatment while maintaining the positive inotropic effects.

## Supporting information

S1 Fig Heart weight and lung weight normalized to tibia length in ISO-treated mice. (A) Heart Weight/Tibia Length (HW/TL) and (B) Lung Weight/Tibia Length (LW/TL) ratios in Sham (control), ‘ISO on’, ‘ISO off short’ and ‘ISO off long’ mice. Sham (control): n = 6; ‘ISO on’: n = 6; ‘ISO off short’: n = 5; ‘ISO off long’: n = 5. Data are presented as mean ± SD. *P < 0.05; **P < 0.01; ***P < 0.001; ****P < 0.0001.
(TIF)

S2 Fig Cardiac fibrosis in ISO-treated mice. (A) Representative Sirius Red stained cardiac sections of Sham (control), ‘ISO on’, ‘ISO off short’ and ‘ISO off long’ mice and (B) quantification of the total fibrotic area in % in cardiac sections at the endpoint of the study. Sham (control): n = 6; ‘ISO on’: n = 6; ‘ISO off short’: n = 5; ‘ISO off long’: n = 5. Data are presented as mean ± SD.
(TIF)

S3 Fig Fractional shortening in ISO-treated mice. Echocardiographic measurement of left ventricular Fractional Shortening (FS) at the endpoint and in the time course of the study in Sham (control), ‘ISO on’, ‘ISO off short’ and ‘ISO off long’ mice. Transthoracic echocardiography was performed on day 0 (pre pump implantation) 14, 26, 28 in all mice and on day 33 in ‘ISO off long’ and respective control mice. Sham (control): n = 6; ‘ISO on’: n = 6; ‘ISO off short’: n = 5; ‘ISO off long’: n = 5. Data are presented as mean ± SD. *P < 0.05; **P < 0.01; ***P < 0.001; ****P < 0.0001.
(TIF)

S4 Fig Pressure volume measurements in ISO-treated mice. (A) Cardiac contractility measured as dP/dt_max_ (mmHg/sec), (B) Cardiac Output (μl/min), (C) Ejection Fraction (EF) in %, (D) Tau Weiss (ms) and (E) Mean Heart Rate (HR) in beats per minute (bpm) in Sham (control), ‘ISO on’ and ‘ISO off short’ mice at day 28. Sham (control): n = 8; ‘ISO on’: n = 5; ‘ISO off’: n = 4. Data are presented as mean ± SD. *P < 0.05; **P < 0.01.
(TIF)

S5 Fig Uncropped images of immunoblots shown in Fig 5. Original images of uncropped immunoblots using antibodies against (A) pPLN-Ser16, (B) pPLN-Thr17 (C) total-PLN and (D) beta-tubulin. The frames indicate cropped immunoblots presented in Fig 5.
(TIF)

S6 Fig Uncropped image of immunoblot shown in Fig 6. Original image of uncropped immunoblot using antibody against HDAC4. The frame indicates cropped immunoblot presented in Fig 6.
(TIF)
