# Supplementary figures and images for: Adaptive versus maladaptive cardiac remodelling in response to sustained β-adrenergic stimulation in a new ‘ISO on/off model’
Source: PLoS One. 2021 Jun 17;16(6):e0248933. doi: 10.1371/journal.pone.0248933 (PMC8211211; doi:10.1371/journal.pone.0248933)

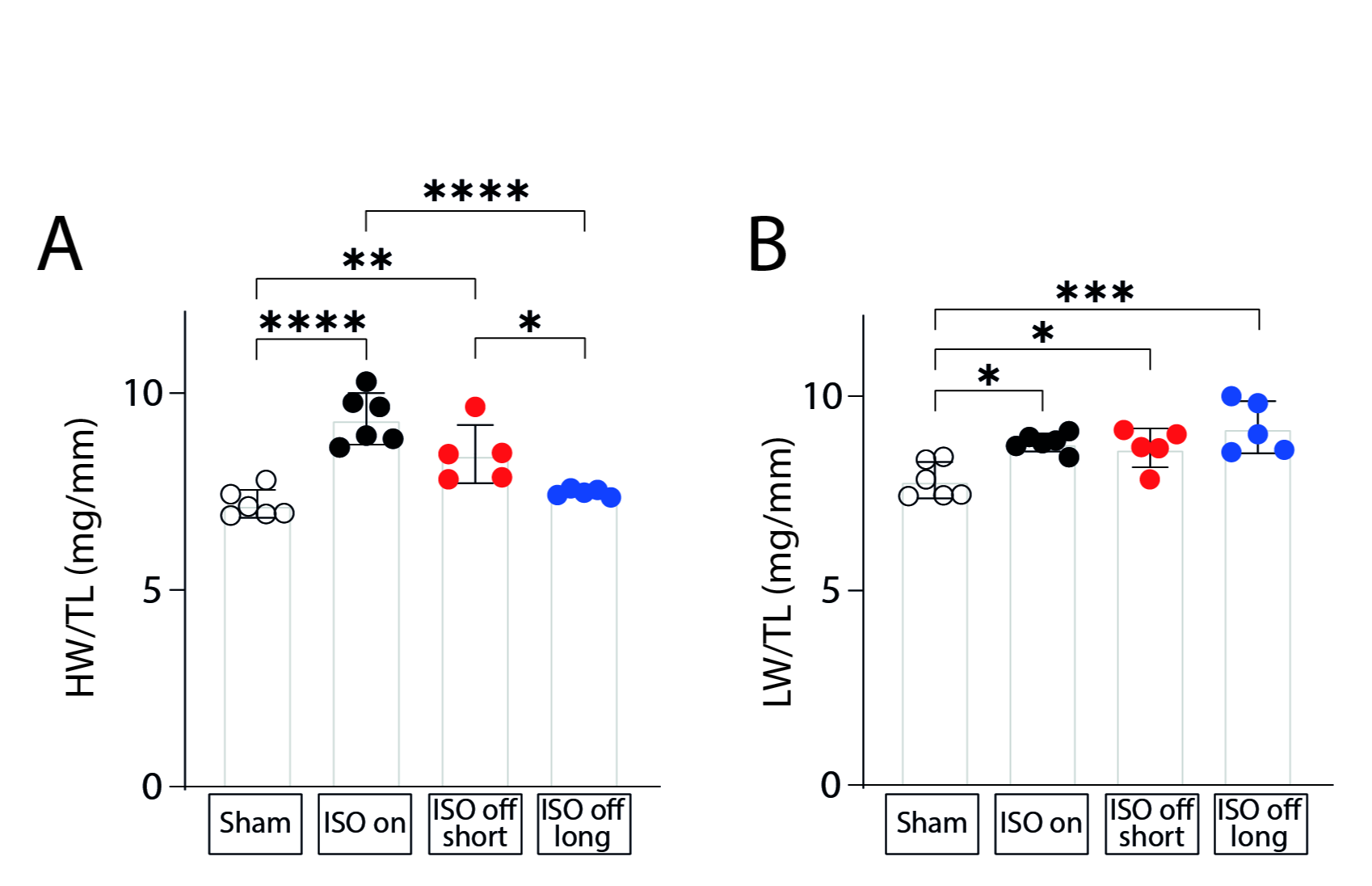

Supplement: S1 Fig — (A) Heart Weight/Tibia Length (HW/TL) and (B) Lung Weight/Tibia Length (LW/TL) ratios in Sham (control), ‘ISO on’, ‘ISO off short’ and ‘ISO off long’ mice. Sham (control): n = 6; ‘ISO on’: n = 6; ‘ISO off short’: n = 5; ‘ISO off long’: n = 5. Data are presented as mean ± SD. *P < 0.05; **P < 0.01; ***P < 0.001; ****P < 0.0001. (TIF) [file pone.0248933.s001.tif]

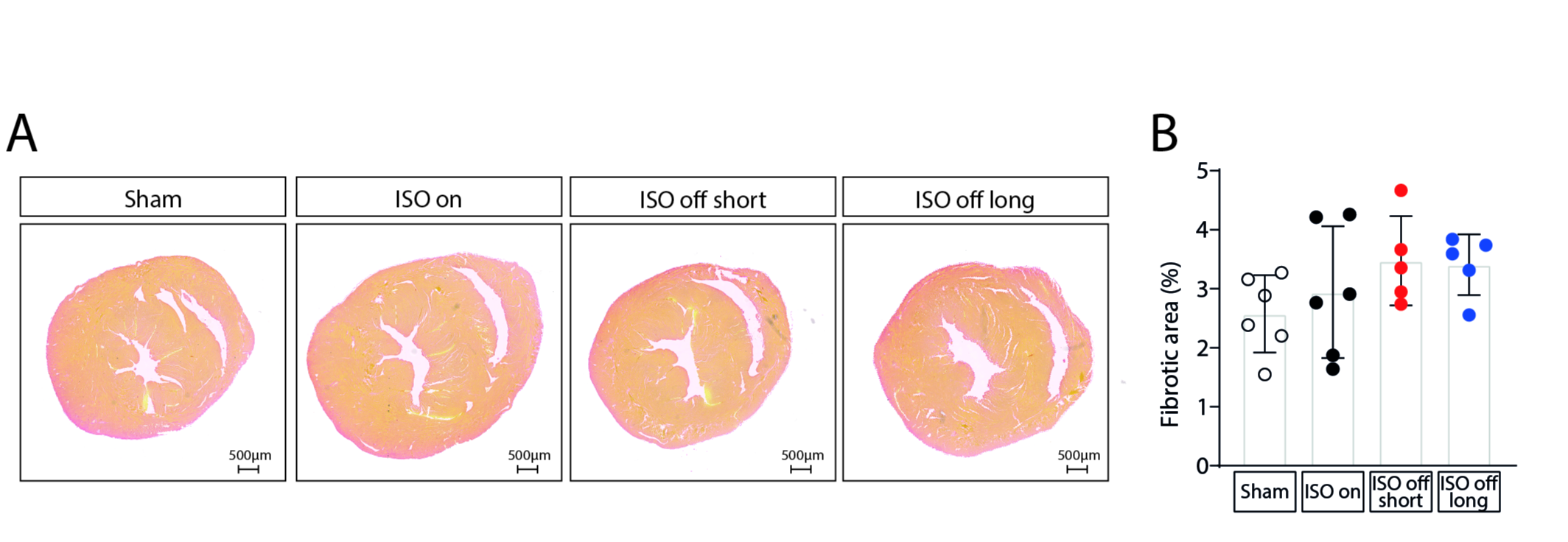

Supplement: S2 Fig — (A) Representative Sirius Red stained cardiac sections of Sham (control), ‘ISO on’, ‘ISO off short’ and ‘ISO off long’ mice and (B) quantification of the total fibrotic area in % in cardiac sections at the endpoint of the study. Sham (control): n = 6; ‘ISO on’: n = 6; ‘ISO off short’: n = 5; ‘ISO off long’: n = 5. Data are presented as mean ± SD. (TIF) [file pone.0248933.s002.tif]

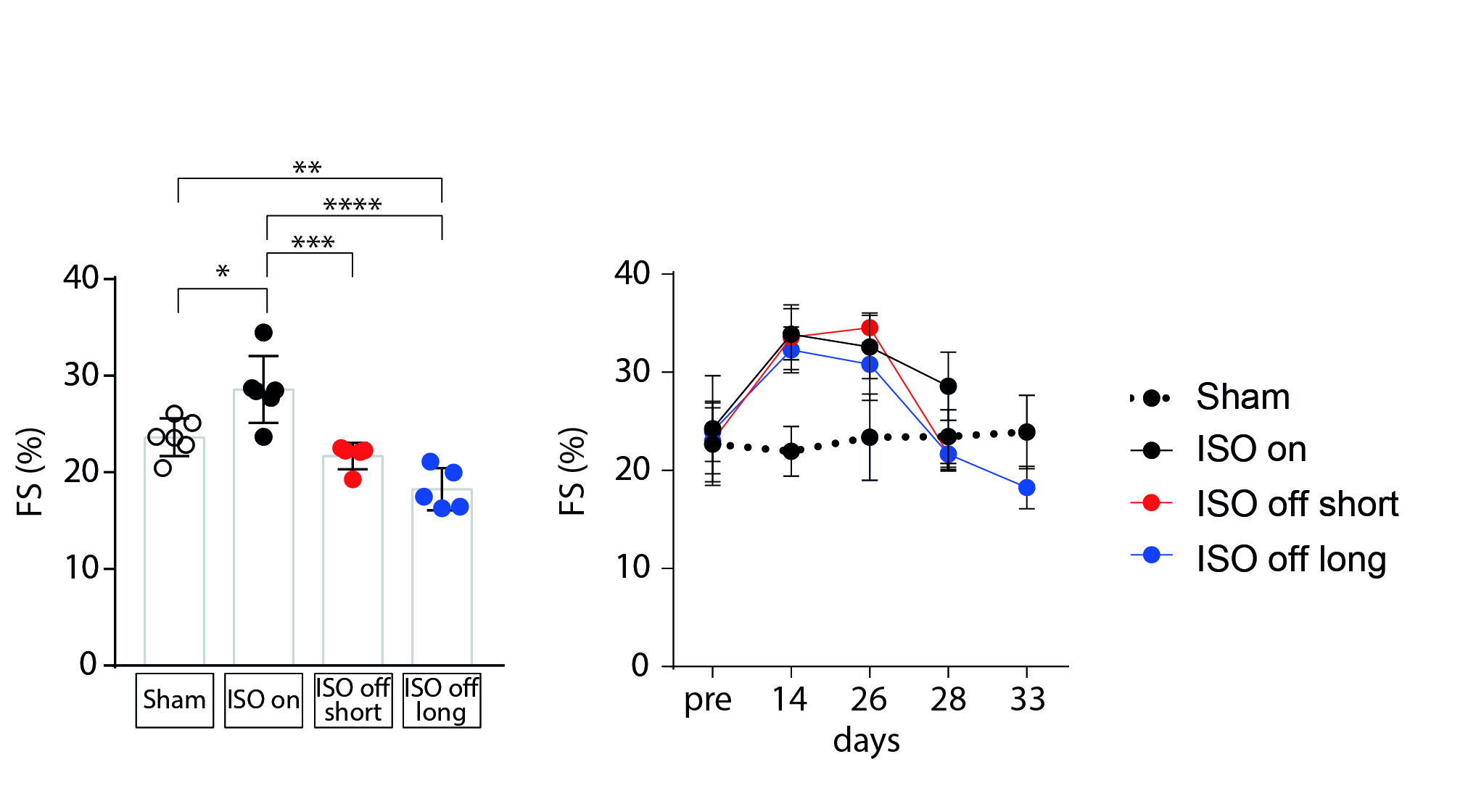

Supplement: S3 Fig — Echocardiographic measurement of left ventricular Fractional Shortening (FS) at the endpoint and in the time course of the study in Sham (control), ‘ISO on’, ‘ISO off short’ and ‘ISO off long’ mice. Transthoracic echocardiography was performed on day 0 (pre pump implantation) 14, 26, 28 in all mice and on day 33 in ‘ISO off long’ and respective control mice. Sham (control): n = 6; ‘ISO on’: n = 6; ‘ISO off short’: n = 5; ‘ISO off long’: n = 5. Data are presented as mean ± SD. *P < 0.05; **P < 0.01; ***P < 0.001; ****P < 0.0001. (TIF) [file pone.0248933.s003.tif]

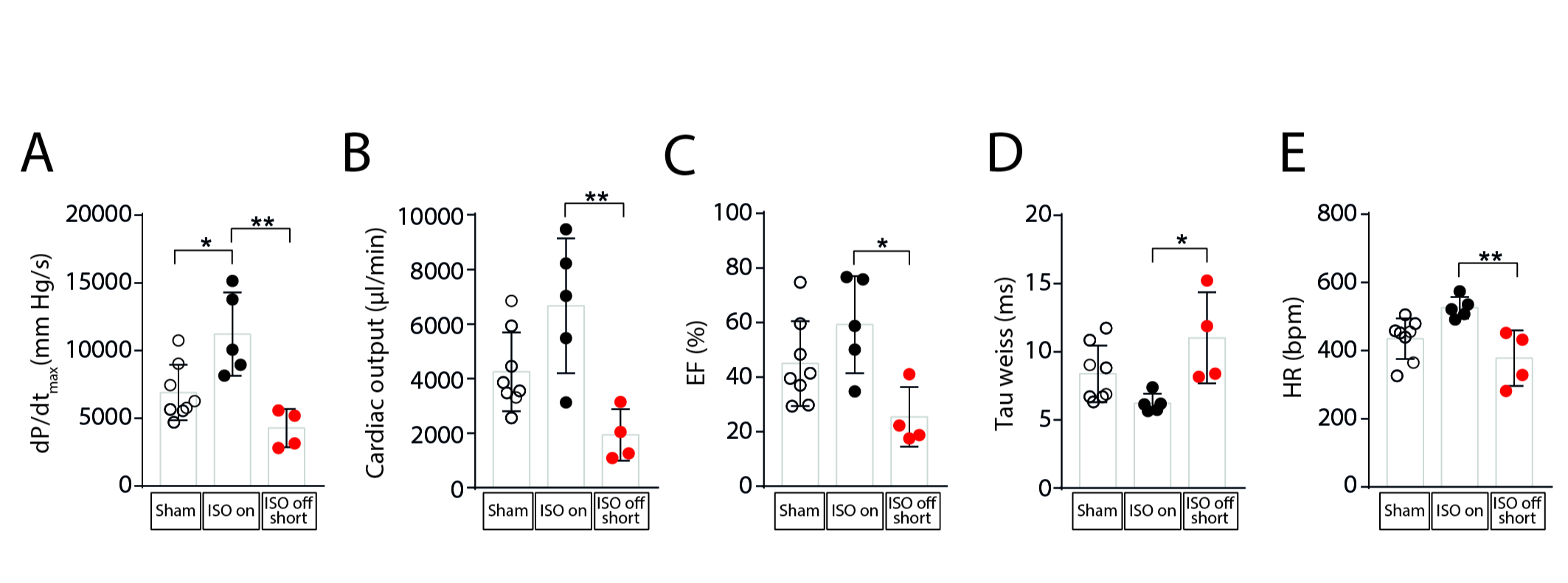

Supplement: S4 Fig — (A) Cardiac contractility measured as dP/dtmax (mmHg/sec), (B) Cardiac Output (μl/min), (C) Ejection Fraction (EF) in %, (D) Tau Weiss (ms) and (E) Mean Heart Rate (HR) in beats per minute (bpm) in Sham (control), ‘ISO on’ and ‘ISO off short’ mice at day 28. Sham (control): n = 8; ‘ISO on’: n = 5; ‘ISO off’: n = 4. Data are presented as mean ± SD. *P < 0.05; **P < 0.01. (TIF) [file pone.0248933.s004.tif]

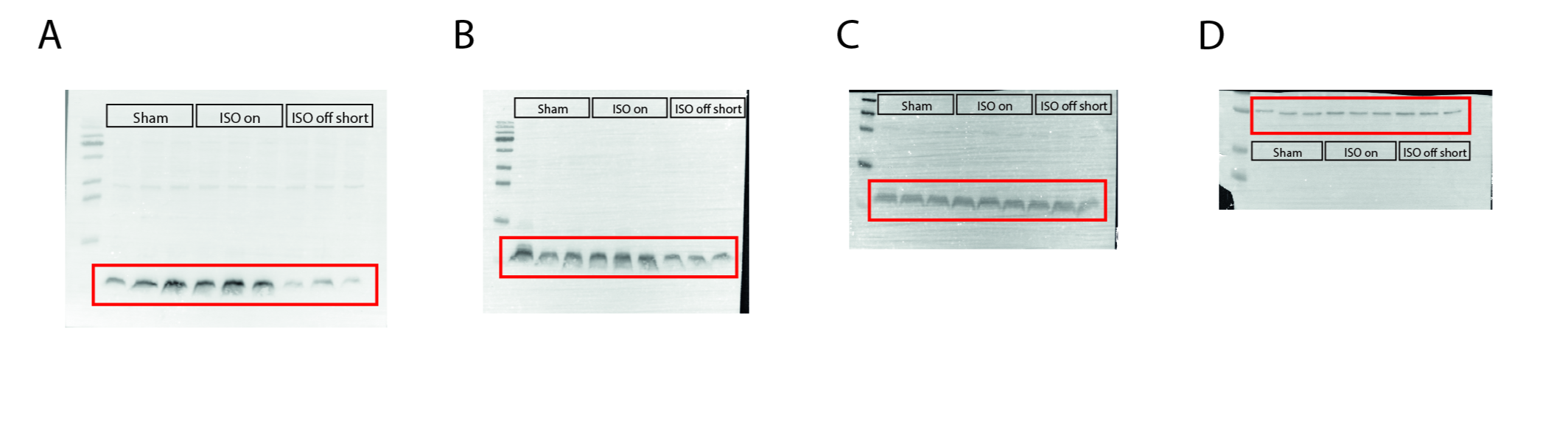

Supplement: S5 Fig — Original images of uncropped immunoblots using antibodies against (A) pPLN-Ser16, (B) pPLN-Thr17 (C) total-PLN and (D) beta-tubulin. The frames indicate cropped immunoblots presented in Fig 5. (TIF) [file pone.0248933.s005.tif]

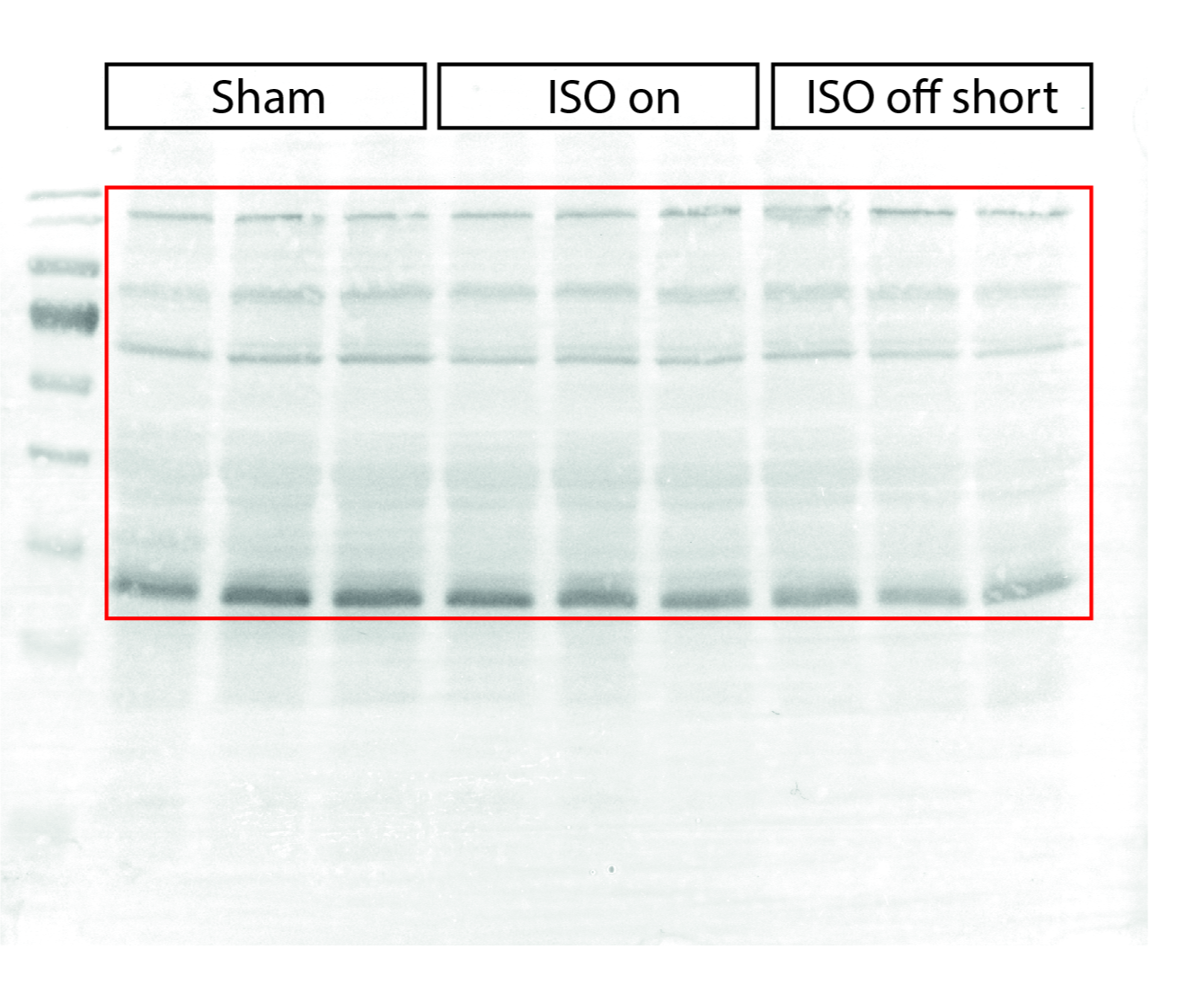

Supplement: S6 Fig — Original image of uncropped immunoblot using antibody against HDAC4. The frame indicates cropped immunoblot presented in Fig 6. (TIF) [file pone.0248933.s006.tif]
